# Clinical and neuroimaging correlates among cohorts of cerebral arteriostenosis, venostenosis and arterio-venous stenosis

**DOI:** 10.18632/aging.102511

**Published:** 2019-12-02

**Authors:** Jiayue Ding, Jingwei Guan, Gary Rajah, David Dornbos III, Weili Li, Zhongao Wang, Yuchuan Ding, Xunming Ji, Ran Meng

**Affiliations:** 1 Department of Neurology, Xuanwu Hospital, Capital Medical University, Beijing, China; 2 Advanced Center of Stroke, Beijing Institute for Brain Disorders, Beijing, China; 3 Department of China-America Institute of Neuroscience, Xuanwu Hospital, Capital Medical University, Beijing, China; 4 Department of Neurosurgery, Jacobs School of Medicine and Biomedical Sciences, University at Buffalo, Buffalo, NY 14203, USA; 5 Department of Neurosurgery, Gates Vascular Institute at Kaleida Health, Buffalo, NY 14203, USA; 6 Department of Neurological Surgery, Semmes-Murphey Clinic and the University of Tennessee Health Science Center, Memphis, TN 38103, USA; 7 Department of Neurosurgery, Wayne State University School of Medicine, Detroit, MI 48201, USA; 8 Department of Neurosurgery, Xuanwu Hospital, Capital Medical University, Beijing, China

**Keywords:** stenosis, white matter lesions, perfusion, metabolism

## Abstract

The purpose of this study was to discriminate the clinical and imaging correlates of cerebral arterial stenosis (CAS), venous stenosis (CVS) and arterio-venous stenosis (CAVS) in the clinical setting. Patients were classified into three groups: CAS (n = 75), CVS (n=74) and CAVS (n=67). Focal neurological deficits were the prominent presenting symptoms in CAS group, while venous turbulence related symptoms were common in both CVS and CAVS group. Risk factor analysis showed the OR (95%CI) for diabetes, male gender and age in CAS vs. CVS group were 13.67(2.71, 68.85), 6.69(2.39, 18.67) and 1.07(1.03, 1.12) respectively. Male gender, diabetes and age in CAVS vs. CAS groups were 0.27(0.11, 0.63), 0.26(0.10, 0.67) and 1.09(1.04, 1.14) respectively, while age in CAVS vs. CVS group was 1.11(1.07, 1.15). The white matter lesions (WMLs) in CAS group varied in size, with clear boundaries asymmetrically distributed in bilateral hemispheres. CVS-induced WMLs revealed a bilaterally symmetric, cloudy-like appearance. The cerebral perfusion was asymmetrically reduced in CAS but symmetrically reduced in CVS group. The clinical characteristics and neuroimaging presentations were different among patients with CAS, CVS and CAVS. We recommended for aged patients, both arterial and venous imaging should be considered in diagnosis of cerebral stenotic vascular disorders.

## INTRODUCTION

Cerebral arterial stenosis (CAS) plays an important role in chronic cerebral circulation insufficiency (CCCI) [1–3]. Persistent reduced cerebral blood volume and flow cause ischemia and hypoxia in the brain tissue, leading to various brain dysfunctions [1]. Chronic cerebrospinal venous insufficiency (CCSVI) has also been confirmed to contribute to neurological deficits and impose a significant impact on cerebral arterial circulation to some extent [4–9]. In theory, CCSVI plays a causative role in pathogenesis of CCCI as well. Venous outflow disturbance may raise the pressure in arterio-venous anastomoses and affect the blood flow (CBF) and volume (CBV) in the arterial system subsequently [4–6]. Advanced neuroimaging can confirm vasculopathic diagnoses, but clinicians often neglect to further explore the disorders as they related to venous vasculopathy. This leaves many patients with rather severe venous stenosis-related ailment untreated and in a great deal of suffering.

Cerebral venous stenosis (CVS) mainly results from extracranial venostenosis (internal jugular vein stenosis, IJVS) and intracranial venostenosis (cerebral venous sinus stenosis, CVSS) [4–7]. Previous publications have described its typical clinical manifestations such as headache, noise, visual impair, sleep disorder and dysphrenia [4, 8]. It can be confirmed by magnetic resonance venography (MRV), computed tomography venography (CTV) and digital subtraction venography (DSV) generally, however, the presentations of brain tissue and perfusion-metabolism status are not fully known. Furthermore, since covered by CAS, some venous stenosis may be misdiagnosed. As for these patients, only treating on arterial stenosis is far from adequate, in contrast, restoring the patency of venous outflow is the key to relieve the refractory neurological symptoms. The aim of this study is to describe the clinical characteristics and imaging findings in CAS, CVS and CAVS, in attempt to further aid the differentiation of these disorders in the clinical settings.

## RESULTS

### Population demographics

**Figure 1:**
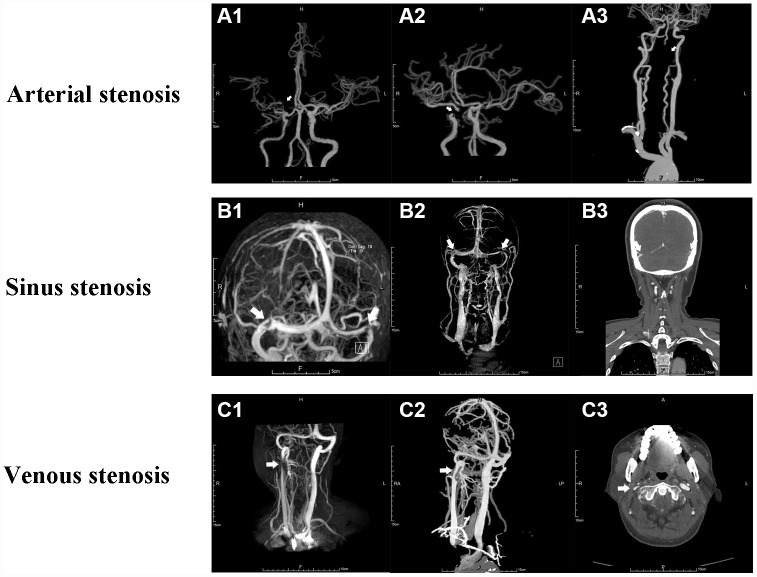
**Angiography of cerebral arterial and venous stenosis.** (**A1**–**A3**) Typical CAS represented by right MCA stenosis, right ICA stenosis and left VA stenosis on CTA respectively. (**B1**–**B3**) Bilateral cerebral transverse sinus stenosis presented on MRV and CTV. (**C1-C3**) Right IJVS J3 segment stenosis presented on MRV and CTV. The stenoses are indicated by arrows.

According to the inclusion and exclusion criteria, a total of 216 subjects who had an average age of 55.0±13.5 years, with 112 male and 104 female, were consecutively enrolled in this study. Seventy five out of 216 patients who had CAS alone were classified into CAS group, 74 patients who had CVS alone were into CVS group, and 67 patients who were confirmed as CAVS made up the CAVS group. The average age of patients was 56.00±9.30, 46.50±14.96 and 63.22±9.87 years respectively (p<0.001). Male gender accounted for 74.7%, 39.2% and 40.3% in each group with a statistical significance as well (p<0.001). The details aforementioned are presented in Table 1 and the representative vascular images are shown in Figure 1.

**Table 1:** Characteristics of enrolled patients.

	**A group**	**V group**	**A-V group**	**P-value**
Demographic				
Num. of patients	75	74	67	-
Age, years	56.00±9.30	46.50±14.96	63.22±9.87	<0.001
Gender, male/female	56/19	29/45	27/40	<0.001
Clinical presentations (%)				
Focal neurological deficits	37 (49.3)	5 (6.8)	18(26.9)	<0.001
Paresthesia	20 (26.7)	7 (9.5)	13 (19.4)	0.025
Sleep disturbances	28 (37.3)	49 (66.2)	47 (70.1)	<0.001
Hearing disorder	4 (5.3)	25 (33.8)	31 (46.3)	<0.001
Visual disorder	2 (2.7)	26 (35.1)	23 (34.3)	<0.001
Headache	15 (20.0)	39 (52.7)	31 (46.3)	0.004
Tinnitus	5 (6.7)	42 (56.8)	41 (61.2)	<0.001
Tinnitus cerebri	3 (4.0)	43 (58.1)	41 (61.2)	<0.001
Dry or puffy eyes	4 (5.3)	34 (45.9)	31 (46.3)	<0.001
Neck discomfort	9 (12.0)	27 (36.5)	19 (28.4)	0.002
Vertigo	34 (45.3)	11 (14.9)	7 (10.4)	<0.001
Dizziness	13 (17.3)	33 (44.6)	36 (53.7)	<0.001
Anxiety or depression	4 (5.3)	14 (18.9)	8 (11.9)	0.032
Nausea or vomiting	8 (10.7)	16 (21.6)	10 (14.9)	0.181
Subjective memory decline	11 (14.7)	8 (10.8)	5 (7.5)	0.393
History (%)				
Diabetes	28 (37.3)	2 (2.7)	13 (19.4)	<0.001
Hypertension	43 (57.3)	16 (21.6)	28 (41.8)	<0.001
Dyslipidaemia	19 (25.3)	28 (37.8)	26 (38.8)	0.158
Hyperhomocysteinaemia	3 (4.0)	5 (6.8)	4 (6.0)	0.752
Hyperuricaemia	3 (4.0)	4 (5.4)	6 (9.0)	0.447
Smoking	26 (34.7)	9 (12.2)	9 (13.4)	0.001
Drinking	22 (29.3)	10 (13.5)	9 (13.4)	0.018

### Location of stenosis

There were a total of 320 arteries having stenosis (173 in CAS versus 147 in CAVS group, p=0.617), including 13 ACA (4 vs. 9, p=0.107), 98 MCA (77 vs. 21, p<0.001), 19 PCA (13 vs. 6, p=0.166), 139 ICA (48 vs. 91, p<0.001) and 51 BA/VA (31 vs. 20, p=0.232); A total of 251 veins were stenotic (130 in CVS versus 121 in CAVS group, p=0.769), including transverse sinus or transverse-sigmoid sinus boundary (42 vs. 31, p=0.315) and IJV (88 vs. 90, p=0.181).

### Clinical features and history

The clinical symptoms differed among the three groups. Focal neurological deficits (hemiplegia, aphasia, dysarthria, etc.), parethesia and vertigo were prominent in CAS group, followed by CAVS and CVS group. On the contrary, other neurological impairments including sleep disturbance, hearing disorder, visual disorder, headache, tinnitus, tinnitus cerebri, dry or puffy eyes, neck discomfort, dizziness, anxiety or depression and nausea or vomiting were commonly seen in CVS and CAVS group. The incidences of subjective memory decline were almost same among three groups.

A history of diabetes, hypertension and smoking was more common in CAS group, when compared with CVS and CAVS groups (all p≤0.001). Other recorded history such as dyslipidaemia, hyperhomocysteinaemia, hyperuricaemia and drinking did not reach a significant difference among three groups (all p>0.05). See Table 1 for complete data entries.

### Risk factor analysis

A pairwise comparison for risk factors using univariate, multivariate and Lasso regression model is shown in Table 2. Univariate analysis indicated that diabetes, hypertension, drinking, smoking, male and old age carried a higher risk of CAS than CVS. The CAS versus CVS group on logistic regression revealed only diabetes [OR(95%CI), 14.40(2.85, 72.67)], male [8.72(2.80, 27.19)] and old age [1.08(1.04, 1.12)] were the predisposing factors to CAS. Lasso regression model was used to prevent multi-collinearity and variable ‘drinking’ was removed from the model at last. Consequently, OR(95%CI) for diabetes, male and old age was 13.67(2.71, 68.85), 6.69(2.39, 18.67) and 1.07(1.03, 1.12) respectively. As regard to CAVS versus CAS group, the differences of diabetes, drinking, smoking, male and age reached significance in univariate analysis. Logistic model uncovered lower prevalence of male gender [OR(95%CI), 0.25(0.10, 0.63)] and diabetes [0.25(0.10, 0.66)] while older age [1.09(1.04, 1.14)] in CAVS group in comparison with CAS group. Lasso analysis deleted variable ‘drinking’ from the model and showed significant OR(95%CI) for male, diabetes and age as 0.27(0.11, 0.63), 0.26(0.10, 0.67) and 1.09(1.04, 1.14) respectively. Univariate analysis revealed diabetes, hypertension and age favored CAVS group compared with CVS group. Nevertheless, only age [OR(95%CI), 1.11(1.06, 1.15)] was strongly associated with CAVS in multivariate Continue variables were presented as mean±standard deviate; Category variables were expressed as n (%). The comparison among three groups were performed using Chi-square test.

**Table 2:** Univariate, multivariate and Lasso analysis for risk factors.

**OR [95%CI]**	**A group versus V group**			**A-V group versus A group**			**A-V group versus V group**		
	**Univariate**	**Multivariate**	**Lasso**	**Univariate**	**Multivariate**	**Lasso**	**Univariate**	**Multivariate**	**Lasso**
Age	9.50 [5.49, 13.51]^**^	1.08 [1.04, 1.12]^**^	1.07 [1.03, 1.12]^**^	7.22 [4.06, 10.38]^*^	1.09 [1.04, 1.14]^**^	1.09 [1.04, 1.14]^**^	16.72 [12.57, 20.87]^*^	1.11 [1.06, 1.15]^**^	1.11 [1.07, 1.15]^**^
Male	4.57 [2.27, 9.20]^**^	8.72 [2.80, 27.19]^**^	6.69 [2.39, 18.67]^**^	0.23 [0.11, 0.47]^*^	0.25 [0.10, 0.63]^**^	0.27 [0.11, 0.63]^**^	1.05 [0.53, 2.06]	-	-
Diabetes	21.45 [4.88, 94.30]^**^	14.40 [2.85, 72.67]^**^	13.67 [2.71, 68.85]^**^	0.40 [0.19, 0.87]^*^	0.25 [0.10, 0.66]^**^	0.26 [0.10, 0.67]^**^	8.67 [1.88, 40.02]^*^	3.78 [0.68, 21.03]	3.94 [0.73, 21.2]
Hypertension	4.87 [2.38, 9.99]^**^	2.23 [0.88, 5.65]	2.11 [0.84, 5.32]	0.53 [0.27, 1.04]	-	-	2.60 [1.25, 5.43]^*^	1.10 [0.45, 2.70]	-
Smoking	3.83 [1.65, 8.91]^*^	1.74 [0.53, 5.76]	1.41 [0.45, 4.42]	0.29 [0.13, 0.68]^*^	0.55 [0.18, 1.73]	0.61 [0.22, 1.70]	1.12 [0.42, 3.01]	-	-
Drinking	2.66 [1.16, 6.10]^*^	0.46 [0.13, 1.60]	-	0.37 [0.16, 0.88]^*^	1.27 [0.39, 4.20]	-	0.99 [0.38, 2.61]	-	-
Dyslipidaemia	0.56 [0.28, 1.12]	-	-	1.97 [0.91, 3.82]	-	-	1.04 [0.53, 2.06]	-	-
Hyperhomo- cysteinaemia	0.58 [0.13, 2.50]	-	-	1.52 [0.33, 7.07]	-	-	0.88 [0.23, 3.41]	-	-
Hyperuricaemia	0.73 [0.16, 3.38]	-	-	2.36 [0.57, 9.34]	-	-	1.72 [0.46, 6.39]	-	-

Univariate analysis used Chi-square test or t-test. Multi-variate analysis was performed through logistic regression model or linear regression model. Lass regression model was used to rule out the multicollinearity effect. All of the results were presented as OR(95%CI).^*^p<0.05; ^**^p<0.01.

analysis. Lasso regression rejected variable ‘hypertension’ and OR (95%CI) for age in this model was 1.11(1.07, 1.15), while the remaining variable ‘diabetes’ did not reach significance.

### Imaging measurement

**Figure 2:**
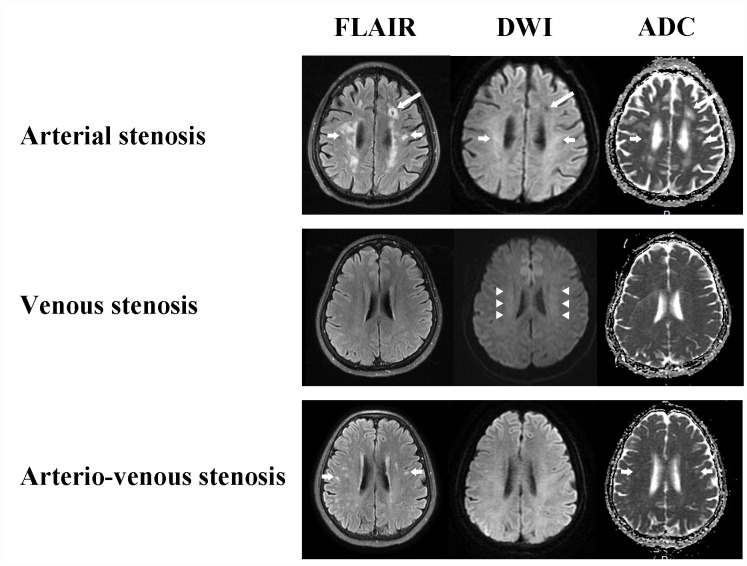
**White matter changes of CAS, CVS and CAVS on MRI maps.** Arterial stenosis always causes cavity (long arrows) and non-symmetrical multiple round, ovoid, patch and fused hyperintensity lesions with clear boundaries (short arrows). Venous stenosis conduces bilateral and symmetrical cloudy-like white matter hyperintensity surrounding ventricles and centrum semiovale (arrow heads). Arterio-venous stenosis also has non-symmetrical focal white matter lesions (short arrows).

The features of WMLs in CAS group were non-symmetrical, point shape, clear boundaries, and varying size lesions, on the contrary, venostenosis-induced WMLs showed bilateral and symmetrical cloudy-like appearance surrounding bilateral ventricles and centrum semiovale. The median (IQR) of Scheltans scales for WMLs were 0.0 (0.0, 0.0) in CVS group, 5.0 (2.0, 8.0) in CAS group, 3.0 (2.0, 4.0) in CAVS group respectively. The scale in CAS group was significantly higher than that in both CAVS and CVS group (p=0.004 and <0.001). Likewise, WMLs in CAVS group were more severe than that in CVS group (p<0.001). The representative images of WMLs in three groups are shown in Figure 2.

**Figure 3:**
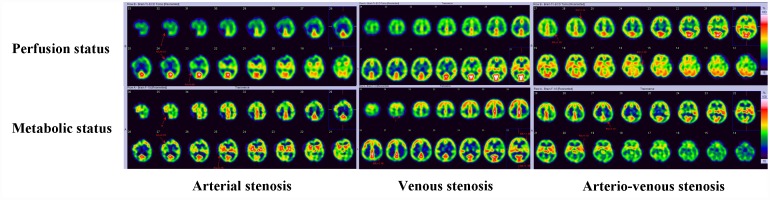
**Perfusion and metabolic status of CAS, CVS and CAVS on SPECT.** CAS and CAVS always has focal or unilateral reduced perfusion with elevated or reduced metabolic status. CVS displays with bilateral symmetrical perfusion reduction and metabolic status maintaining normal or elevation frequently.

A total of 37 patients completed SPECT assessment, including 12 in CAS group, 12 in CVS group and 13 in CAVS group. The frontal and parietal lobe were regarded as ROI in this study because all of the involved patients had profound perfusion reduction in these areas. The perfusion and metabolic status were abnormal in these patients. The perfusion and metabolism mismatch was always noticed in CVS group (68.3%), followed by CAS group (41.6%) and CAVS group (30.8%). The radio difference of regional perfusion between two sides of ROI on SPECT was substantially higher in CAS group than that in CVS group [median (IQR), 0.18 (0.12, 0.25) vs. 0.0 (0.0, 0.14), p=0.003], while no statistical differences were seen between CAVS group [0.11 (0.0, 0.24)] and any other two groups (all p>0.05). There was a tendency for higher radio difference of glucose metabolism in ROI in CAS group than CVS group [0.26 (0.15, 0.75) vs. 0.18 (0.10, 0.23), p=0.089], while when compared with CAVS group [0.25 (0.13, 0.31)], the difference was small (all p>0.05). A typical presentation of perfusion and metabolism difference among three groups is shown in Figure 3.

## DISCUSSION

A total of 216 patients were enrolled in this study with nearly even sample size among the three groups. We selected the subjects from two prospective registered trials rather than one cohort, therefore, this study is not a cross-sectional epidemiological investigation, but a nested case-control study. Only patients with NIHSS≤3 or TIA were involved in order to be well matched for the presentations of CCCI [4, 8, 10]. Minor disability induced by CAS can be confused with the symptoms from CVS, whereby this cohort is warranted to be distinguished.

In this study, patients with CAS alone commonly presented with focal neurological deficits (hemiplegia, aphasia, dysarthria, etc.), parethesia and vertigo, in contrast to those with CVS alone and CAVS, in whom non-specific whole brain symptoms and venous turbulence related symptoms dominated the clinical presentation. Venous outflow obstruction does not destroy focal brain tissue, rather rendering the whole brain to a low-perfusion background through the indirect impact on CBF and CBV on capillary pressures [4–8]. On the contrary, arterial stenosis often reduces the regional perfusion leading to focal brain tissue impairment [11–13]. When the functional area is invaded, the focal symptoms occur finally [13]. Given most of the patients in CAVS group were derived from the cohort whose primary diagnosis was venous disorders (82%), this seemed to impart a significant selection bias into the results toward a low prevalence of focal deficits in these participants. Intracranial hypertension induced by venous stenosis may cause headache, visual disorder and vomiting, and the outflow turbulence is associated with tinnitus and abnormal noise, all of which are the specific presentations in CCSVI entities [4–8].

Multivariate analysis showed patients in CAVS group had the highest age, followed by CAS group and CVS group. In order to remove the multi-collinearity function, Lasso regression was used and to the same conclusion, that is, elderly patients should be considered of having arterial comorbid with venous stenosis pictures. Young people are vulnerable to cerebral venous disorders due to the high possibility of immune disease, genetic disease, gestation and so forth, and aged people tend to be more prone to CAS due to atherosclerosis formation [13, 14]. Patients with CAVS have a higher age than those with CAS or CVS alone, possibly suggesting a time dependent process or later manifestation of the disease. Degenerative cervical spondylosis and severe atherosclerosis inducing arterial dilation may compress venous outflow patterns in these population [4, 8, 15, 16]. The ratio of patients with male gender and diabetes were significantly higher in CAS group than the other two groups after Lasso regression analysis. Females were more susceptible to cerebral venous system diseases due to the specific physiological status [14].

Previous studies report CCSVI is associated with severe WMLs is to some degree at odds with our findings [17, 18]. The ischemia-induced demyelination derived from CAS displays non-symmetrical multiple round, ovoid, patch or even fused lesions while CVS typically presents with symmetrical cloudy-like appearance surrounding bilateral ventricles and centrum semiovale [4, 7, 19, 20]. CAVS has the concomitant imaging characteristics of arterial and venous stenosis. Multiple sclerosis (MS) was considered a relative of CCSVI, however, in this study, none of patients had convincing evidence to support an MS diagnosis [21–23]. Through SPECT imaging, we noted a perfusion-metabolism mismatch could be seen more frequently in the CVS group, followed by CAVS group and finally the CAS group. Long-term low-perfusion induced by CCCI is implicated in the propensity of the brain tissue metabolism rate to increase so as to suffice the cellular basic need [1]. As perfusion decreases further, the compensatory mechanisms cannot protect cells from ischemia, and as a result, the necrose or appoptose ultimately reducing metabolism. Our results indicated that both CAS and CVS can contribute to CCCI status. Regional circulation disturbances lead to focal perfusion-metabolism anomalies while venous outflow obstruction may cause the whole brain perfusion-metabolism deficiency [9]. This explains why focal signs typically occurred in the CAS group, and non-focal symptoms were more often seen in the CVS group.

Currently CVS can be corrected by stenting. Venous stenting is conducted for patients with a pressure gradient >8mmHg after failed conservative treatment (weight loss, antithrombosis, diamox) and balloon dilation. A large number of studies have demonstrated the safety and efficacy of stenting in cerebral venous outflow disturbance. Hence, as for CAVS, especially in patients with intractable non-focal neurological symptoms, stenting in stenotic venous segments may be a plausible therapy [24, 25].

There are some limitations to this study. (1) We enrolled patients from two cohorts that one is with primary diagnosis of CAS and one is with CVS. Most of the patients (82%) with CAVS were from the second cohort, which means, despite with arterial and venous disorders concurrently, the initial diagnosis of CVS indicated venous disorder symptoms dominated the majority of clinical presentations in these patients. (2) As aforementioned, this is a nested case-control study rather than cross-section study. The presentation percentage cannot be used as epidemic data for these populations due to high selection bias. (3) DSA is confirmed as the gold standard for vascular stenosis. Although the imaging techniques in this study can detect most of the stenosis accurately, some false imaging diagnosis is inevitable. (4) The vertebral veins and the azygous vein also play an important role in CCSVI, however, we only enrolled patients with definite IJVS and CVSS in this study. (5) There were lots of confounding factors (such as holding breath, breathing in and out, etc.) influencing the venous stenosis extent evaluation. The venous flow is much more variable and can be influenced by neck manipulation and breathing patterns, which may bias our results. The changes of venous flow in different breathing condition will be further investigated in future studies.

## CONCLUSIONS

CAS typically presents with focal neurological deficits, while CVS displays non-specific symptoms such as intracranial hypertension and venous outflow turbulence induced disturbance related symptoms. Symptoms in CAVS were complex, which could comprise CAS and CVS related clinical manifestations. Both cerebral arterial and venous stenosis can result in CCCI. Aged patients might have CAS and CVS concomitantly. Venous imaging should also be conducted in these patients even though the diagnosis of arterial stenosis is present. Considering limitations of this study, further epidemic investigations with large sample size are needed in the future.

## MATERIALS AND METHODS

### Participants

This study was designed as a nested case-control study utilizing a prospectively maintained data set and has been approved by the Institutional Ethic Committee of Xuanwu Hospital, Capital Medical University (Beijing, China) in accordance with the guidelines of the 1964 Declaration of Helsinki. Any specific interventions or tests were performed only after informed consent was obtained from the patients. From October 2017 through March 2019, a total of 247 subjects who were diagnosed with CAS [including intracranial and extracranial arterial stenosis (ICAS and ECAS)] and/or CVS (including IJVS and CVSS) in the departments of Neurology and Neurosurgery of Xuanwu Hospital, Capital Medical University, were enrolled in this study. Inclusion criteria included the severity of venous disorder, only patients with National Institute of Health Stroke Scale (NIHSS) ≤3 were involved [10]. Intracranial / extracranial included arteries comprised the anterior cerebral artery (ACA), middle cerebral artery (MCA), posterior cerebral artery (PCA), internal carotid artery (ICA), basilar artery (BA) and vertebral artery (VA). Venous anatomy sites included all segments of cerebral venous sinus (such as transverse sinus, sigmoid sinus, sagittal sinus, etc.), and extracranial veins such as the internal jugular vein (IJV). The diagnosis of CAS was confirmed by magnetic resonance angiography (MRA) and/or computed tomographic angiography (CTA) and/or high-resolution magnetic resonance imaging (HR-MRI), and CVS was determined by MRV and/or CTV and/or HR-MRI. CAS referred to ACA, MCA, PCA, BA, VA or ICA ≥50% stenosis confirmed by MRA or CTA and/or HR-MRI. Patients should have focal or non-focal neurological deficits. CVS referred to IJV or cerebral venous sinus narrowing of ≥50% in respect to the proximal adjacent vein segment, as presented in MRV, CTV or HR-MRI. Meanwhile, for IJVS, at least one abnormal collateral vessel ≥50% of the maximal diameter of the adjacent IJV or at least two abnormal collateral vessels <50% of the maximal diameter of the adjacent IJV had to be present. Abnormal collateral circulation generation indicated severe venous outflow insufficiency when venous stenosis existed, in contrast, CVS cannot be confirmed if lack of abnormal collateral vessels were not present, regardless of vein narrowing [15, 26–28]. Magnetic resonance imaging (MRI), including axial T1-, T2-weighted sequence, diffuse weighted image (DWI) and fluid attenuated inversion recovery (FLAIR) weighted sequence were performed in each enrolled patient. Single-photon emission computed tomography (SPECT) was conducted in a portion of patients to assess the perfusion and glucose metabolism status as well. According to the invaded vessels, the enrolled patients were divided into the groups of CAS, CVS and CAVS.

All of the patients in the database were screened according to inclusion and exclusion criteria presented as following.

Inclusion criteria: (1) age range from 18 to 80 years; (2) definite radiographic diagnosis of ECAS/ICAS/IJVS/ CVSS; (3) afflicted by focal or non-focal neurological symptoms; (4) NIHSS≤3 or transient ischemic attack (TIA).

Exclusion criteria: (1) comorbid with other life-threatening diseases; (2) severe cerebral hemorrhage, infarction and abnormal lesions (such as tumors); (3) with a history of cerebral vascular surgery; (4) poor compliance.

### Imaging protocols

All participants underwent MRI with 3-tesla (MAGNETOM Verio, Siemens Healthcare, Erlangen, Germany) and a standard 32-channel head coil. Typical MRI sequence parameters included T1: repetition time (TR)/echo time (TE)=160/3.1ms, field of view (FOV)=240×240mm^2^; T2: TR/TE=3800/93ms, FOV=240×240mm^2^; FLAIR: TR/TE=8000/94ms, FOV=218×240mm^2^; DWI: TR/TE=5500/90ms, FOV=240×240mm^2^. Some performed time-of-flight (TOF) MRA: TR/TE=22/4ms; FOV=170×170mm^2^, MRV: TR/TE=3.4/1.3ms; FOV=170×170 mm^2^ and HR-MRI: TR/TE=800/22ms; FOV=160×200mm^2^. All CTA/CTV were conducted using a 64-row detector CT scanner, Discovery HD 750 (GE Healthcare, Milwaukee, Wisconsin, USA) with an administration of iodinated contrast (Iopromide 370; Bayer, Germany). Regional cerebral blood flow was evaluated with Technetium-99m ethylene cysteine dimer (^99m^Tc-ECD) and metabolism status was detected with fluorodeoxyglucose (^18^F-FDG) on SPECT. SPECT scanning was conducted at 30 minutes after IV ^99m^Tc-ECD (25 mCi) bolus injection and 40 minutes after ^18^F-FDG bolus injection. Original images were reconstructed as perfusion maps in a 128×128 matrix and presented in coronal, axial, and sagittal planes by the Butterworth filtering function. All participants underwent imaging in the supine position.

### Assessment

The demographics, clinical manifestations and history were recorded entirely at admission. Meanwhile, the detailed angiography presentations on MRA, CTA, MRV and CTV were depicted. The white matter lesions (WMLs) were evaluated on MRI through semiquantitative Scheltans scales with a range from 0–84, where scores 0–6 were given in the periventricular regions, 0–24 in the deep white matter, 0–24 in the infratentorial regions and 0–30 in the basal ganglia [29]. We set region of interest (ROI) to describe the regional change of perfusion and metabolism on SPECT maps. The degree of cerebral perfusion and metabolism were categorized on a 6-point scale according; to the chromatic aberration change (white 100%, red 80%, yellow 60%, green 40%, blue 20% and black 0%) [4]. The white and red represented normality, yellow and green were reduction while blue and black indicated robust reduction or even disappearance. Both regional perfusion and metabolism status on SPECT were also represented as R/L, a radio of right ROI radionuclide uptake versus left (^99m^Tc-ECD for perfusion and ^18^F-FDG for metabolism) [30, 31]. The degree of symmetry of right and left perfusion or metabolism for ROI was evaluated by radio difference [the absolute value of (R/L minus 1)], that is, the closer to 0 it was, the more symmetrical the bilateral perfusion or metabolism was. A perfusion and metabolism mismatch indicated regions with decreased ^99m^Tc-ECD uptake indexes in combination with elevated or normal ^18^F-FDG uptake indexes. Two senior neuro-radiologists who were blinded to the clinical database evaluated all of the images in a side-by-side fashion. A third assessor would re-examined the images if inconsistencies occurred between the two assessors. The final decision was in accordance with the ratings that had the majority, at least two thirds votes.

### Statistical analysis

R software (http://www.r-project.org) was used to perform the analysis. Continuous data was complied with a Gaussian distribution and was presented as mean±standard deviation (SD), otherwise as median (interquartile range, IQR). Categorical data was expressed as counts (percents, %). Student’s t test and Mann-Whitney U tests were used to compare continuous variables, and the Pearson χ^2^ test or Fisher’s exact tests were used to compare categorical variables among these groups. Only risk factors that are closely related to the dependent variables (with p<0.05) were enrolled as variates in the logistic regression model to assess independent risk factors. However, high multicollinearity may amplify the regression co-efficiency, leading to inaccurate results. In order to prevent multi-collinearity, a Lasso regression model was used to remove the independent variables that had high multi-collinearity with other independent variables. A 2-side p value<0.05 was indicative of statistical significance.
